# Railway Overhead Contact System Point Cloud Classification †

**DOI:** 10.3390/s21154961

**Published:** 2021-07-21

**Authors:** Xiao Chen, Zhuang Chen, Guoxiang Liu, Kun Chen, Lu Wang, Wei Xiang, Rui Zhang

**Affiliations:** 1 Faculty of Geoscience and Environmental Engineering, Southwest Jiaotong University, Chengdu 611756, China; XChen_RS@my.swjtu.edu.cn (X.C.); chenzhuang@my.swjtu.edu.cn (Z.C.); rsgxliu@swjtu.edu.cn (G.L.); kunchen_2020@163.com (K.C.); xiangwei@my.swjtu.edu.cn (W.X.); 2 National and Local Joint Engineering Laboratory of Safe Space Information Technology for High-Speed Railway Operation, Southwest Jiaotong University, Chengdu 611756, China; 3 Department of Road and Bridge Engineering, Sichuan Vocational and Technical College of Communications, Chengdu 611130, China; luwang_19870104@163.com

**Keywords:** railway OCS, point cloud, scale adaptive feature algorithm, DBSCAN algorithm, classification

## Abstract

As the railway overhead contact system (OCS) is the key component along the high-speed railway, it is crucial to detect the quality of the OCS. Compared with conventional manual OCS detection, the vehicle-mounted Light Detection and Ranging (LiDAR) technology has advantages such as high efficiency and precision, which can solve the problems of OCS detection difficulty, low efficiency, and high risk. Aiming at the contact cables, return current cables, and catenary cables in the railway vehicle-mounted LiDAR OCS point cloud, this paper used a scale adaptive feature classification algorithm and the DBSCAN (density-based spatial clustering of applications with noise) algorithm considering OCS characteristics to classify the OCS point cloud. Finally, the return current cables, catenary cables, and contact cables in the OCS were accurately classified and extracted. To verify the accuracy of the method presented in this paper, we compared the experimental results of this article with the classification results of TerraSolid, and the classification results were evaluated in terms of four accuracy indicators. According to statistics, the average accuracy of using this method to extract two sets of OCS point clouds is 99.83% and 99.89%, respectively; the average precision is 100% and 99.97%, respectively; the average recall is 99.16% and 99.42%, respectively; and the average overall accuracy is 99.58% and 99.69% respectively, which is overall better than TerraSolid. The experimental results showed that this approach could accurately and quickly extract the complete OCS from the point cloud. It provides a new method for processing railway OCS point clouds and has high engineering application value in railway component detection.

## 1. Introduction

**Figure 1:**
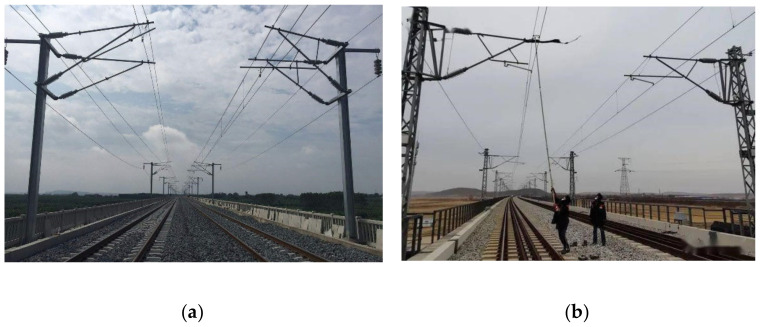
The struct of OCS and manual monitoring method: (**a**) overhead contact system; (**b**) manual monitoring method.

With the rapid development of railway electrification, the reliability and safety of railway traction power equipment is a key research issue [1,2,3]. Among them, the detection of railway overhead contact systems (OCS) is the key link to ensure the normal operation of railway traction electric power. The overhead contact system shown in Figure 1a is the infrastructure of the power supply for trains. Under the influence of pantograph and environmental factors, the components of OCS, such as contact cables, return current cables, and catenary cables, are prone to deformation. These deformations may cause instability of the power supply, affect the durability of components, and even cause accidents [4]. Therefore, the regular detection of OCS to ensure its stability is of great significance for the normal operation and safety of the railway system. Although the conventional manual monitoring method shown in Figure 1b is more flexible, there are many shortcomings in artificial detection such as low efficiency, high risk, and human factors such as personnel experience, which can affect detection results. Therefore, it has been unable to meet the existing detection needs [5,6,7,8]. At present, determining the detection method with high efficiency, high precision, and low risk has become a research hotspot.

The research on the high-efficiency detection work of the OCS started relatively early. Kusumi et al. [9] developed an OCS inspection system that can be installed on rail vehicles. Liu et al. [10] described a wireless system for monitoring a railway’s signaling, control, or infrastructure condition. In 2012, the Ministry of Railways of China proposed a ‘High-speed Railway Power Supply Security Detection and Monitoring System,’ named the ‘6C’ system [4]. However, due to the limitations of the accuracy of sensors, equipment size, and corresponding algorithms, it is difficult to achieve the desired effect in railways.

With the rapid development of detection technology, the Light Detection, and Ranging (LiDAR) measurement system has become an effective method for solving these problems. Manual inspection requires operators to carry measuring instruments for measurement in a limited skylight period; generally, the efficiency is low and influenced by subjective factors and environmental factors, and there are many hidden dangers on site, which brings many problems to prevention and control. Compared with the conventional methods, LiDAR technology has the advantages of high detection efficiency, good accuracy, non-contact, and being less affected by environmental factors [11,12]. LiDAR systems can quickly and accurately obtain a large number of 3D point clouds and extract valuable spatial data with details that cannot be achieved by previous patrol detection techniques [13]. It has a unique advantage in the acquisition of 3D information of railways in complex and even dangerous areas and has a great application prospect in the detection of OCS [14,15]. Therefore, the classification of OCS from the point cloud is an effective method for OCS detection [16,17]. Due to the cost and special environmental conditions of the railway, it is not suitable to obtain point clouds using airborne LiDAR and drones, which are widely used to obtain OCS point clouds. Therefore, vehicle-mounted LiDAR is the best choice to obtain OCS point clouds. Scholars have done some research on the theory and algorithm of extracting OCS and other facilities from point clouds containing environmental factors. Mostafa [18] developed an automated method to recognize railroad infrastructure from 3D LiDAR data. The proposed methodology recognizes key components of the railroad corridor based on their physical shape, geometrical properties, and the topological relationships among them. Zhang et al. [19] used principal component analysis combined with information entropy theory to achieve the extraction of power lines, and the method is based on vehicle-mounted LiDAR data and the parallel characteristics of power lines and rails. Jung et al. [20] designed a classifier based on the MrCRF (multi-range Conditional Random Field) and SVM (support vector machine) to identify ten key components introducing neighbor information for the misclassification caused by similar features. The MrCRF only takes spatial information within limited ranges and does not consider the shape and size variance. Arastounia [21] identified contact cables by applying a region growing algorithm and then classified catenary cables according to the positional relationship between the contact cables and catenary cables. Sánchez-Rodríguez et al. [14] presented a method for automatically inspecting the clearance gauge and the deflection of the aerial contact line in railway tunnels. Ariyachandra and Brilakis [22] detected lines using RANSAC and classified cables based on the heights of the lines relative to the track structure. Gutiérrez-Fernández et al. [23] presented a new method for the automatic extraction of power cable locations in railways using surface LiDAR systems. Lin et al. and [7] presented a method based on deep learning to recognize point clouds of OCS components. In general, the mean extraction accuracy of these methods can reach 92–98%. At present, the extraction of OCS from point clouds mainly relies on the structural characteristics of the OCS and their positional relationship with the track; the clustering method is mainly used. This process will bring more errors, and it is necessary to set thresholds when extracting the trackbed and the OCS, which is more subjective. For catenary cables with large curvatures, the effect is often poor. Recently, deep convolutional neural networks for point cloud segmentation [24,25] have been brought into being, but this method is less used in the extraction of OCS. The point clouds of the catenary are large-scale, and a few hundred meters of railway scene can contain more than 100,000 points. Therefore, it is quite hard to train a good segmentation model with whole scene data, which requires high memory and training time costs. There are also researchers using voxel-based, patch context analysis, improved PCA (principal component analysis), improved region growing method, and other methods to achieve effective large-scale point cloud classification [26,27,28,29,30,31]. However, for such a long and narrow point cloud set, to achieve precise extraction and classification, these methods may require further research. Increasing the amount of LiDAR can effectively increase the perception range of visual systems and the density of point clouds [32,33] while bringing problems of point cloud splicing, camera calibration, data volume, and cost.

This paper aims to develop algorithms to reduce the subjective selection process of parameters, extract and make full use of the spatial distribution characteristics of the target points and improve the accuracy of classification extraction. We adopted a scale adaptive feature classification algorithm and the DBSCAN (Density-Based Spatial Clustering of Applications with Noise) algorithm considering OCS characteristics to classify the target point cloud in this paper. The scale adaptive feature classification algorithm and entropy eigenvalue were adopted to judge the distribution of geometric structure features of the point cloud in 3D space realized rough classification of OCS point cloud to maximize the retention of the target point cloud and reduce the calculation time and complexity of fine classification. We introduced the entropy eigenvalue to reduce the difficulty of parameter selection in the rough classification process. Then, the DBSCAN algorithm was utilized to classify OCS precisely.

## 2. Methodology

To complete the classification task of OCS, this section adopted a coarse to fine classification method. Firstly, the non-ground point cloud is roughly classified to maximize the retention of the target point cloud, reduce the calculation time and complexity of fine classification, and improve the accuracy of fine classification. Furthermore, the target point cloud is classified more accurately based on the rough classification. This section focuses on the scales adaptive feature classification algorithm and uses the algorithm for the fine classification of non-ground point clouds.

### 2.1. Removing Ground Points

Before the OCS classification, the ground points in the point cloud need to be removed, and we use the Euclidean distance clustering segmentation algorithm [34,35] to achieve this goal. The method refers to the actual distance between points in m-dimensional space. The calculation formula is as follows:

(1) $$dL=\sqrt{\sum_{}^{n}{\left(p-q\right)}^{2}}$$

(2) $$Q=\left\{p,q|dL<r,p,q\in P\right\}$$

where *dL* is the distance between two points, *Q* represents the processed points, and *P* is the 3D point cloud set.

The basic principle of the Euclidean distance clustering segmentation algorithm is to traverse all points in the point cloud as a point clustering center. We then judge the similarity between the remaining points and clustering centers according to the Euclidean distance between the cluster centers and the remaining points. We assign each point to the cluster that is most similar to that point. Lastly, the center of each new cluster is recalculated, and the above process is repeated until the loop center converges and the segmentation of the point cloud can be completed.

### 2.2. Rough Classification

To classify most OCS points in the non-ground point cloud, this paper takes the scale geometric structure features (dimensional features) of the point as the basis of classification. We combine the scale feature classification algorithm [36,37] with the entropy eigenvalue to form a new algorithm called the scale adaptive feature classification algorithm. We use the algorithm to realize the rough classification of the OCS point cloud. Before analyzing the distribution of geometric structure features, the points in the neighborhood of each target point (i.e., target point set) are searched; the selection of the best neighborhood radius is elaborated in Section 2.2.3. Furthermore, the covariance matrix and eigenvalues corresponding to the target point sets are calculated. Secondly, the relationship between the eigenvalues is analyzed, and then the geometric structure distribution characteristics of the target points in 3D are obtained. The contact cable, return current cable, and catenary cable in OCS are linear structure characteristics, so all the data with linear distribution characteristics in the point cloud are retained.

The scale adaptive feature classification algorithm aims at finding the best neighborhood radius of each point and working directly in 3D space to calculate the geometric structure distribution characteristics of the target point in 3D space. The algorithm mainly includes two steps:

1. Traverse the predefined minimum and maximum neighborhood scales in a certain step. The 3D features of points in the different neighborhood radii of the target point are calculated to represent the geometric structure distribution of the point in 3D space, which can be divided into linear features (1D), planar features (2D), and discrete features (3D);
2. Selection of the best neighborhood-scale *r*: when searching for the neighborhood radius, select a neighborhood radius to make one of the dimensional features of the point in the neighborhood more obvious than the other two. By comparing the dimension results of these three features in the range of minimum radius and maximum radius, the best radius is determined.

In the process of obtaining geometric structure features, the research focuses on calculating the dimension features contained in the neighborhood of the target point. The neighborhood points $v_{P}^{r}$ are all the points surrounded by the sphere, with the target point P as the center and the scale r as the radius. $P_{k}$ is a set of points that need to be verified, the mathematical expression is as follows:

(3) $$P_{k}\in v_{P}^{r}\Leftrightarrow \left\|P-P_{k}\right\|\leq r$$

Therefore, the 3D sphere is used to define the neighborhood shape. The definition method is isotropic and rotation invariant so that the geometric features are not affected by the shape deviation of the neighborhood, and *r* is the only parameter to be optimized.

#### 2.2.1. Calculation of Eigenvalue

To obtain the three-dimensional feature distribution of the target points in the three-dimensional space, it is necessary to find the *X*, *Y*, and *Z* coordinate values in the point cloud set and calculate the covariance matrix and eigenvalues corresponding to the point cloud set.

First, let $x={\left(x_{1},y_{2},z_{3}\right)}^{T}$, $\bar{X}=\frac{1}{n}\sum_{i=1}^{n}X_{i}$ is the center of gravity of *n* points, $M={\left(X_{1}-\bar{X},X_{2}-\bar{X},\ldots ,X_{n}-\bar{X}\right)}^{T}$, and the corresponding covariance matrix *C* is as follows:

(4) $$C=\frac{1}{n}M^{T}M$$

*C* is the covariance matrix of 3×3 and is a symmetric positive definite matrix.

The eigenvalue decomposition of *C* is expressed as:

(5) $$C=R\Lambda R^{T}$$

According to the eigenvalue decomposition of the covariance matrix, the eigenvalues $\lambda_{1}$, $\lambda_{2}$, and $\lambda_{3}$ ($\lambda_{1}\geq \lambda_{2}\geq \lambda_{3}>0$) can be obtained. Let $\delta_{j}=\sqrt{\lambda_{j}}$, ∀j∈[1,3], denote the standard deviation in the direction of the corresponding feature vector. The PCA algorithm can be used to obtain the three main directions of $v_{P}^{r}$, and the size of the eigenvalues obtained indicates their importance.

#### 2.2.2. Geometric Feature Analysis

Geometric structure features can be judged according to the size of the feature value. The discriminant index [38,39] can be divided into linear features $\partial_{1D}$, planar features $\partial_{2D}$, and discrete features $\partial_{3D}$; the formula is as follows:

(6) $$\partial_{1D}=\frac{\delta_{1}-\delta_{2}}{u}$$

(7) $$\partial_{2D}=\frac{\delta_{2}-\delta_{3}}{u}$$

(8) $$\partial_{3D}=\frac{\delta_{3}}{u}$$

where *u* is the normalization coefficient, when $u=\delta_{1}$ or $u=\sum_{d=1,3}\delta_{d}$, we get $\partial_{1D},\partial_{2D},\partial_{3D}\in [0,1]$. To simplify the calculation and expression, we take u to be $\delta_{1}$, then $\partial_{1D}+\partial_{2D}+\partial_{3D}=1$.

When $\delta_{1}\gg \delta_{2},\delta_{3}≃0$ and the value of $\alpha_{1D}$ is greater than the value of $\alpha_{2D}$ and $\alpha_{3D}$, the point cloud set represents a linear feature distribution. If $\delta_{1},\delta_{2}\gg \delta_{3}≃0$ and the value of $\alpha_{2D}$ is greater than the value of $\alpha_{1D}$ and $\alpha_{3D}$, the point cloud set represents a planar feature distribution. When $\delta_{1}≃\delta_{2}≃\delta_{3}$ and the value of $\alpha_{3D}$ is greater than the value of $\alpha_{1D}$ and $\alpha_{2D}$, the point cloud set represents a discrete feature distribution.

#### 2.2.3. Selection of Scale

**Figure 2:**
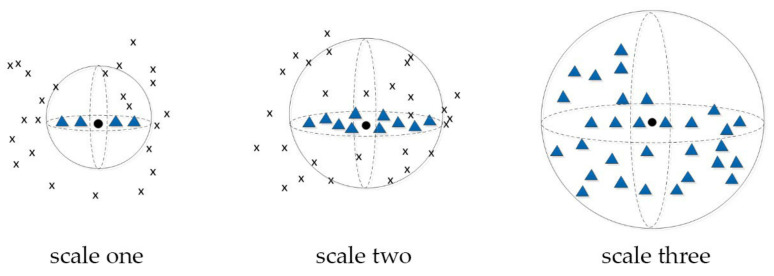
Neighborhood of point cloud at different scales.

Before analyzing the geometric structure of the point, it is necessary to determine the *r*. We set multiple radii *r* so that the target point shows different geometric structure distribution characteristics under different *r* [40,41]. By changing the value of *r*, we construct a spherical space neighborhood of different sizes with the target point as the center, thus forming a scale space. The neighborhood distributions of point clouds in different scale spaces are shown in Figure 2, in which the blue triangular points represent the points in the neighborhood.

In the first scale, the sample data represent linear features, namely one-dimensional features; in the second scale, the sample data represent planar features, namely two-dimensional (2D) features, and in the third scale, the sample data represent discrete features, namely three-dimensional (3D) features.

To determine the best neighborhood scale, the upper and lower bounds $r_{\min}$ and $r_{\max}$ of scale *r* are determined. The setting $r_{\min}$ depends on the noise of the point cloud, the specifications of the sensor, and the constraints of the calculation. The $r_{\max}$ can be directly selected according to the scale of the largest object in the scene.

To choose the best scale, the entropy eigenvalue *E_f_* is introduced. This unpredictable measure is given by the information entropy of the discrete probability distribution $\left\{\alpha_{1D},\alpha_{2D},\alpha_{3D}\right\}$; the entropy of the point cloud set $v_{P}^{r}$ is defined as follows:

(9) $$E_{f}(v_{P}^{r})=-\alpha_{1D}\ln(\alpha_{1D})-\alpha_{2D}\ln(\alpha_{2D})-\alpha_{3D}\ln(\alpha_{3D})$$

When the entropy $E_{f}(v_{P}^{r})$ is smaller, it means that all the features of the point cloud set are more concentrated in one of the dimensions. Then we can get the definition of the best scale $r_{E_{f}}^{*}$:

(10) $$r_{E_{f}}^{*}=\arg\min E_{f}\left(v_{p}^{r}\right),r\in [r_{\min},r_{\max}]$$

Each point in the point cloud can be calculated to obtain different entropy feature values under different neighborhood scales. In order to select the best neighborhood-scale *r* value, we need to traverse all points in the point cloud at different neighborhood scales, calculate the entropy eigenvalues of all points, and obtain an average value of entropy eigenvalues. When the average value of entropy eigenvalues reaches the minimum, the corresponding neighborhood scale *r* is the best scale. 

Then we can get the radius of the most concentrated feature between *r*_min_ and *r*_max_.

### 2.3. Fine Classification

Most non-target points are removed after the rough classification, and then this paper used the DBSCAN algorithm, which takes into account the characteristics of OCS to realize the fine classification of the OCS point cloud. The algorithm combines the traditional DBSCAN algorithm with the spatial feature information of OCS to achieve the final goal of accurate classification.

DBSCAN is a density-based clustering algorithm, which has been widely used in the classification of point clouds. The clustering recognition of this algorithm is not affected by noise points, and the time complexity is low [42,43]. The algorithm includes two steps:

1. Set the values of the neighborhood radius *Eps* and density *MinPts*, select the point *P* that meets the requirements of the two parameters through the distance metric and find all the density-reachable object points from *P* to form a cluster;
2. Repeat step 1 until all the points are processed. The algorithm will be described in detail below.

#### 2.3.1. Determination of Clustering Parameters

Before implementing the DBSCAN algorithm, we need to set reasonable parameters of density *MinPts* and neighborhood radius *Eps* [44] to filter the point cloud.

The value of *Eps* can be obtained by the *k*-dist distance curve [42], and the critical inflection point of the *k*-dist distance curve is the corresponding better *Eps* value. If the value is too small, most of the data can not be clustered; if the value is too large, multiple clusters and most of the objects will be merged into the same cluster. The choice of the value of *k* depends on the user’s experience. Finally, take *k* to be *MinPts*. This method of determining *Eps* based on statistical characteristics of the *k*-dist curve analysis data is called the heuristic method, which is suitable for 2D and 3D data.

For the selection of parameter *MinPts* [45], it is the minimum expected cluster size; that is, a cluster is composed of at least *MinPts* points. As a guiding principle:

(11) MinPts≥dim+1

Among them dim represents the dimension of the data to be clustered.

It is unreasonable to take *MinPts* equal to 1 because when it is set to 1, each independent point is a cluster, and the result is the same as the nearest neighbor of the hierarchy distance. Therefore, *MinPts* must be greater than 3. If the value is too small, the result in the sparse cluster will not be used for further expansion of the class because the density is less than *MinPts*; if the value is too large, two neighboring clusters with higher density may merge into the same cluster. 

After setting the two parameters values of *MinPts* and *Eps*, we take the Euclidean distance as a measure [46,47] to select the data point *P* that meets the requirements of the two parameters.

#### 2.3.2. Principle of DBSCAN Algorithm Considering Railway OCS Characteristics

According to the characteristics of the number of overhead conductors and the constant structure distribution, the contact cable and return current cable are almost in the same plane. The height of the catenary cable is the highest, and it is directly above the contact cable. According to the above characteristics, we use the DBSCAN algorithm that combines the spatial position relationship between cables of OCS to realize the fine classification of OCS cloud data. The principle of the algorithm is as follows:

1. Select the *Eps* value and *MinPts* value according to the method in Section 2.3.1, and perform DBSCAN clustering on OCS point cloud after rough classification.
2. After clustering, the number of points of each cluster is compared and sorted. Because the data volume of contact cable, catenary cable, and return current cable in the point cloud after rough classification is in the top three of the whole data set, the top three point clouds are saved and the remaining data set is deleted.
3. Compare the values in the Z direction in the three saved point clouds. Compare the calculated Z-means of each group of clusters, and the group of clusters with the largest Z-mean value is judged as the catenary cable point cloud.
4. Taking the catenary cable point cloud as the reference object, the Euclidean distance between the remaining two groups of cluster point cloud and catenary cable point cloud on the XOY plane is calculated, respectively. The cluster with a smaller Euclidean distance is the contact cable point cloud, and the cluster with a larger Euclidean distance is the return current cable point cloud.

**Figure 3:**
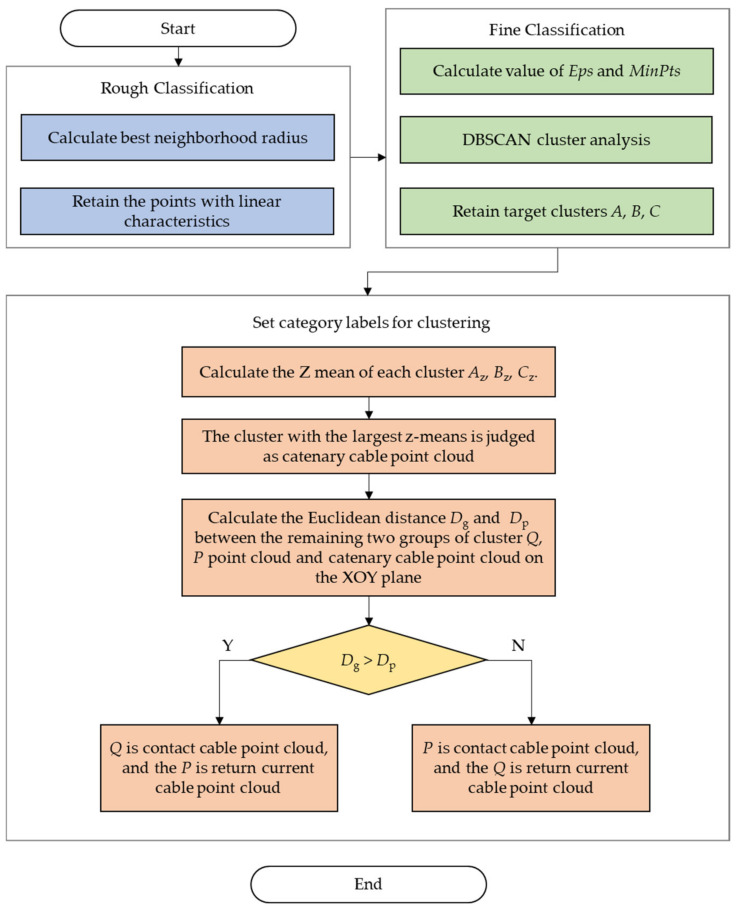
Flow chart of DBSCAN classification algorithm considering OCS characteristics.

The algorithm flow is shown in Figure 3.

## 3. Data Acquisition and Experiment

### 3.1. Experimental Data

**Figure 4:**
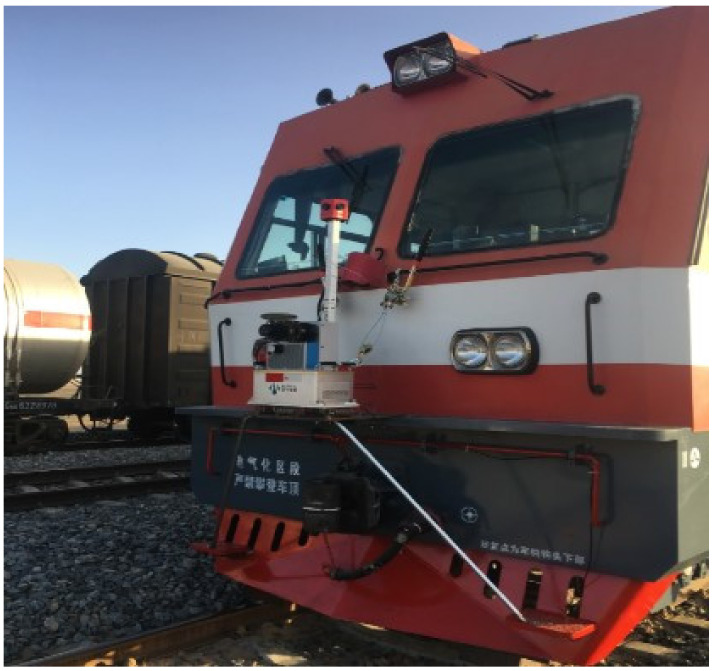
Vehicle-mounted LiDAR and its installation location.

**Figure 5:**
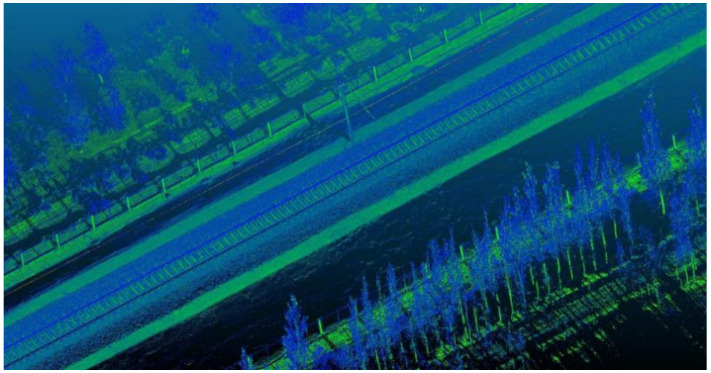
Original point cloud.

**Figure 6:**
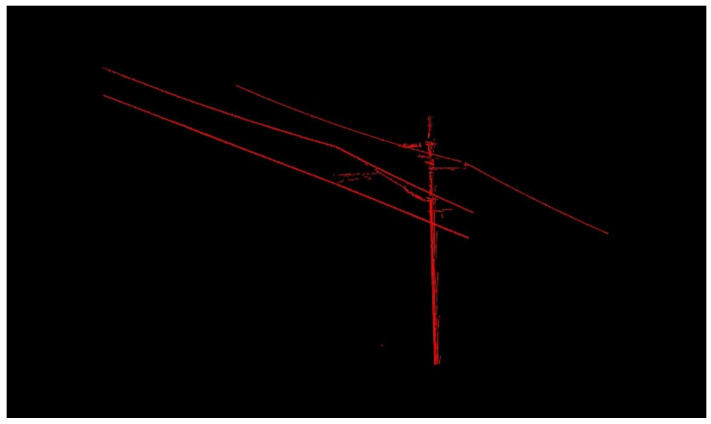
Railway OCS point cloud with a length of 50 m.

**Figure 7:**
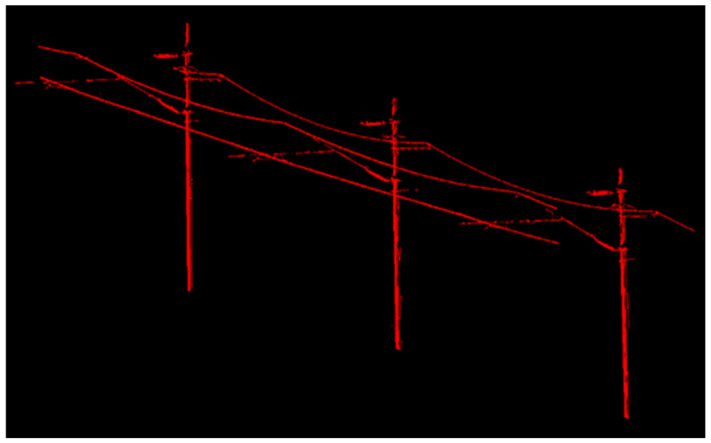
Railway OCS point cloud with a length of 100 m.

The experimental data used in this paper are the point clouds with lengths 100 m and 50 m that are intercepted from the vehicle-borne LiDAR of a domestic railway trunk line. The point cloud was collected by the German Z + F three-dimensional laser scanner profiler 9012; Table 1 shows the technical parameters of the scanner. Figure 4 shows the point cloud collection equipment and its installation location. Euclidean distance clustering was performed on the collected point cloud to obtain a non-ground point cloud, which is the experimental data in this article. Figure 5 shows the original point cloud. Figure 6 and Figure 7 show the experimental data with a length of 50 m and 100 m, respectively. Next, we conducted experiments on these two sets of data separately.

The LiDAR system was installed on the locomotive, roughly located on the vertical plane of the track centerline. The installation of the equipment does not cost too much time. Railway OCS is set above the track; only the ground points need to be removed to get the OCS point cloud with noise points. Ground points can be removed by Euclidean distance clustering. The removal of ground points is relatively simple. Overall, the data used in this article will not require a great amount of upstream work.

### 3.2. Rough Classification

**Figure 8:**
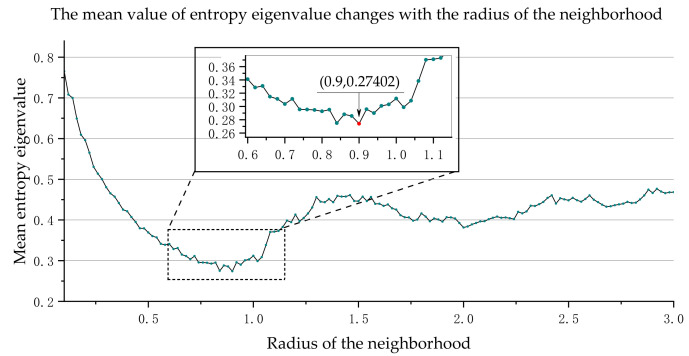
The mean entropy eigenvalue of 50 m OCS point cloud changes with the radius of the neighborhood.

**Figure 9:**
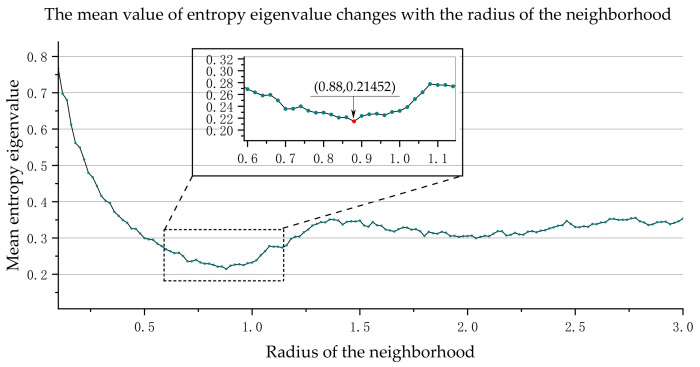
The mean entropy eigenvalue of 100 m OCS point cloud changes with the radius of the neighborhood.

According to the algorithm in Section 2, the feature distribution of the point cloud can be obtained. Firstly, select the scale according to the factors of boundary selection, adjustment accuracy, and calculation time. We sample *r* values between [0.1, 3.0] as multiple spatial scales (i.e., multiple neighborhood radii *r*) for experiments. We choose 0.02 as the iteration step because the scan line spacing of LiDAR is between 0.02 and 0.03. The minimum value of *r* is selected based on experience; usually, five times the distance between scanning lines, and the maximum value of *r* is select based on the largest object. Secondly, we use PCA to calculate the three main directions of $v_{P}^{r}$ in the candidate spatial scale and analyze whether the points are set in the linear distribution (one-dimensional feature). Finally, we retain and output the linear distribution feature point cloud. In order to select the best scale, we calculate the entropy eigenvalue *E_f_* of each point in the point cloud and find the average value of the entropy eigenvalues of all points. When the mean value of the entropy eigenvalue is the smallest, the corresponding scale is the best scale $r_{E_{f}}^{*}$. According to the calculation results, Figure 8 and Figure 9 show the mean entropy eigenvalue of 50 m of OCS data and 100 m of data changes with the radius of the neighborhood, respectively. For the 50 m OCS point cloud, when the neighborhood radius *r* is equal to 0.90, the mean value of entropy eigenvalue reaches the minimum. Therefore, we select 0.90 as the best neighborhood scale. Likewise, we select 0.88 as the best neighborhood scale for the 100 m OCS point cloud.

### 3.3. Fine Classification

Before the classification using the DBSCAN algorithm considering the characteristics of the OCS, the SOR (Statistical Outlier Removal) filter was used to denoise the point cloud after the rough classification, which is essential to further remove part of the non-OCS point cloud.

**Figure 10:**
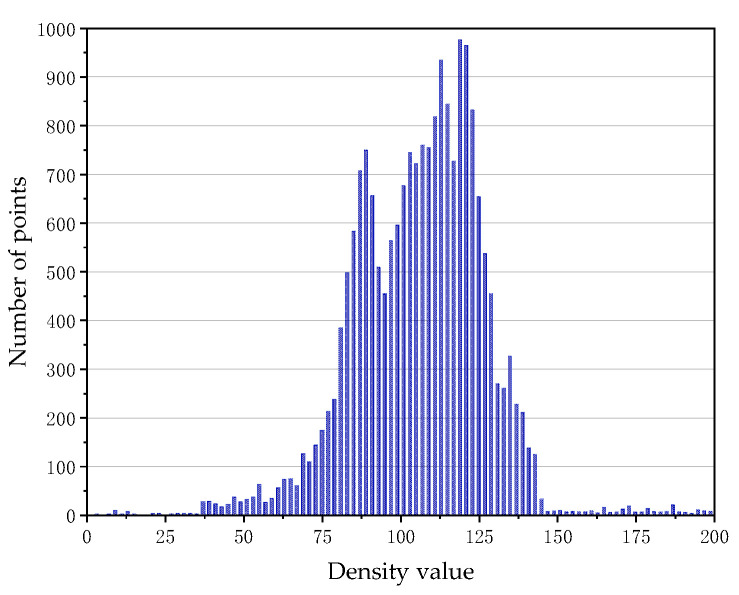
Density histogram of 50 m OCS point cloud.

**Figure 11:**
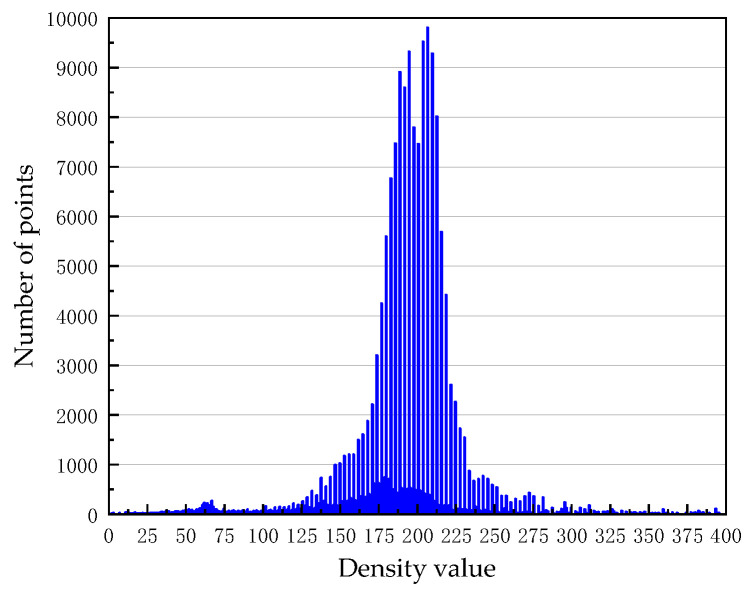
Density histogram of 100 m OCS point cloud.

Firstly, determine the values of *MinPts* and *Eps* according to the clustering parameter determination method in Section 2.2.1, and then the overhead contact system fine classification experiment is carried out. We count the neighborhood density of the point cloud after rough classification. The graph of the number of points changing with the neighborhood density is shown in Figure 10 and Figure 11. As the figures show, most points are concentrated in a certain density range, and we treat the density as valid density ranges. The density ranges of 50 m data and 100 m data are 38–145 and 50–325, respectively. 

**Figure 12:**
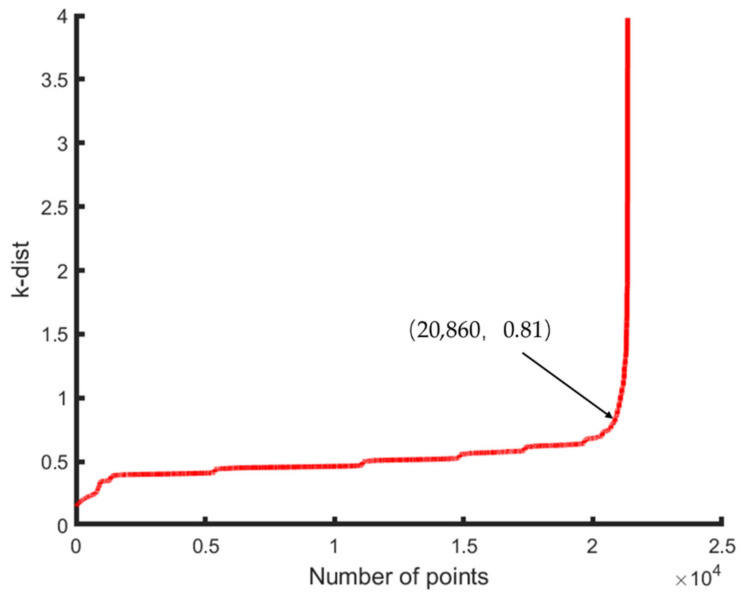
k-dist graph of 50 m OCS point cloud.

**Figure 13:**
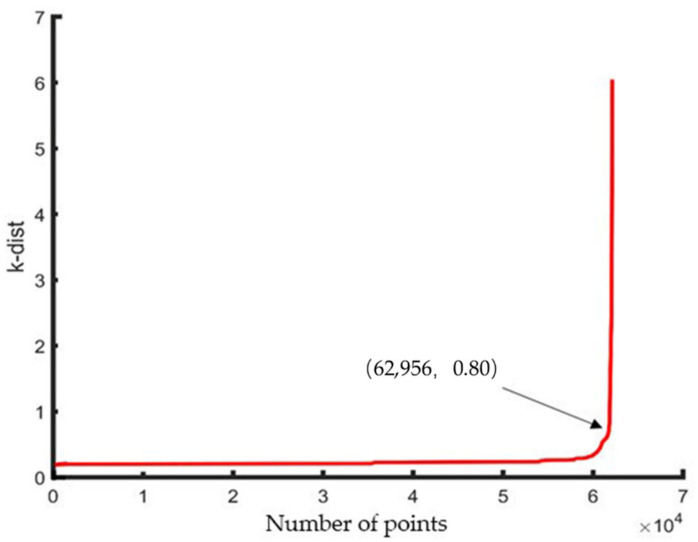
k-dist graph of 100 m OCS point cloud.

Take the smaller point cloud density of the data set as the minimum expected cluster. Take *MinPts* of 50 m point cloud equal to *k*, *k* equal to 38, and then draw the ascending *k*-dist graph through the heuristic method to determine the value of *Eps* according to the information in the graph. Figure 12 shows the *k*-dist graph of the 50 m OCS point cloud. Similarly, for the 100 m data, take *MinPts* equal to 50, and Figure 13 shows the *k*-dist graph of the 100 m OCS point cloud.

In Figure 12 and Figure 13, the abscissa represents the number of points, and the ordinate represents the *k*-dist corresponding to the number of points. Then, by sorting the *k*-dist map, the recessed area, which is the threshold dividing point (critical value), is obtained. Therefore, the *MinPts* of the 50 m OCS point cloud and 100 m OCS point cloud are equal to 38 and 50, respectively. The required *Eps* value is the *k*-dist value corresponding to the threshold point; at this time, *Eps* = 0.8 in the two datasets. After determining the parameter values of *MinPts* and *Eps*, the final OCS fine classification is performed on the experimental data using the DBSCAN algorithm that takes into account the characteristics of the OCS.

## 4. Results and Discussion

### 4.1. Results of Classification

#### 4.1.1. Results of Rough Classification

**Figure 14:**
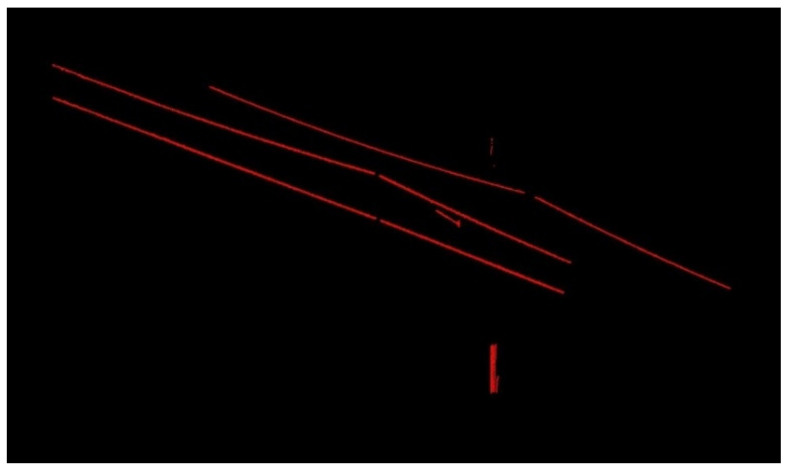
Experimental results of OCS with a length of 50 m.

**Figure 15:**
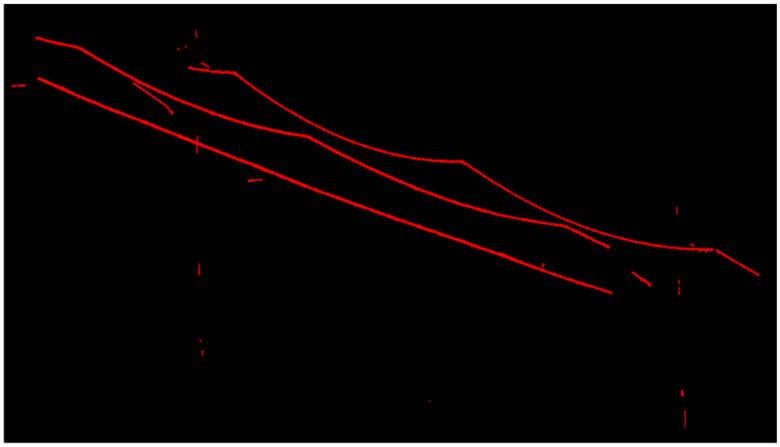
Experimental results of OCS with a length of 100 m.

Due to a large number of points in the large scene, to obtain a more accurate linearly distributed feature point cloud (OCS point cloud), this paper iterated the methods in Section 2.1. The final results of the two sets of point clouds are shown in Figure 14 and Figure 15.

Multiple calculations have significantly reduced the point cloud of the OCS mast and cantilever. Therefore, after many iterations of the experiment, as many OCS points as possible are retained to lay a good data foundation for the subsequent fine classification. As shown in Figure 14 and Figure 15, a large number of points of OCS poles and masts have been eliminated, and a small number of OCS poles and masts with linear distribution characteristics are left. The point cloud of the overhead contact system is almost completely retained.

#### 4.1.2. Results of Fine Classification

**Figure 16:**
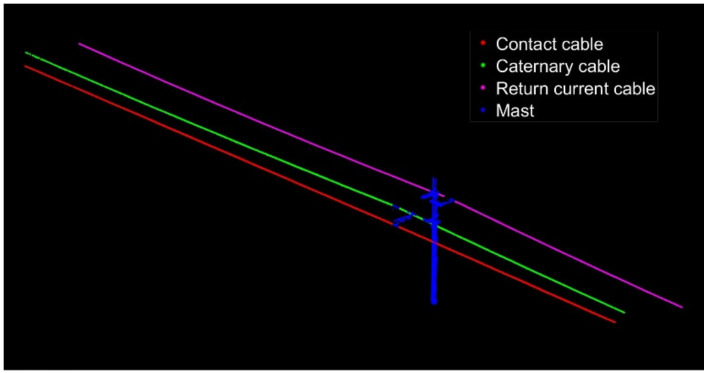
Fine classification results of the OCS point cloud with a length of 50 m.

**Figure 17:**
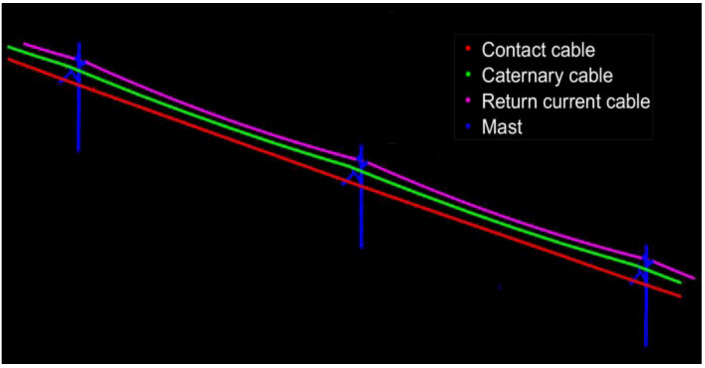
Fine classification results of the OCS point cloud with a length of 100 m.

After determining the parameter values of *MinPts* and *Eps*, the DBSCAN algorithm considering the characteristics of OCS is used to classify the OCS. The red point cloud is classified as a contact cable, the green point cloud is a catenary cable, and the pink point cloud is a return current cable. The previously deleted and unclassified point clouds are merged into a point set as the imprecise and incomplete OCS mast point cloud, which includes some noise points and OCS clouds that had been wrongly deleted. The classification results of the two point clouds are shown in Figure 16 and Figure 17.

### 4.2. Performance Evaluation

In order to evaluate the accuracy of the extraction algorithm of OCS, we use four accuracy evaluation indexes in pattern recognition, namely precision, recall, accuracy, and overall accuracy [47]. To compare and analyze the number of points of the contact cable, return current cable, and catenary cable extracted by the algorithm and the actual points of the three.

(1) Precision: refers to the percentage of the extracted OCS points in the real OCS points. (Real railway OCS points refer to manually classified OCS points, the same below)

(12) $$\mathrm{Precision}=\frac{TP}{TP+FP}\times 100\%$$

(2) Recall: refers to the percentage of the real OCS points judged as railway OCS points in the extracted railway OCS points.

(13) $$\mathrm{Recall}=\frac{TP}{TP+FN}\times 100\%$$

(3) Accuracy: refers to the percentage of the total point cloud judged as the OCS point in the total point cloud.

(14) $$\mathrm{Accuracy}=\frac{TP+TN}{TP+TN+FP+FN}\times 100\%$$

(4) Overall accuracy: as a measure of overall accuracy, it refers to the harmonic mean value of accuracy and recall α=1.

(15) $$\mathrm{Overall}\;\mathrm{accuracy}=\frac{(\alpha^{2}+1)\times \mathrm{Precision}\times \mathrm{Recall}}{\alpha^{2}(\mathrm{Precision}+\mathrm{Recall})}\times 100\%\;$$

where *TP*, *TN*, *FP,* and *FN* denote true positive, true negative, false positive, and false negative, respectively. A more detailed explanation is as follows, *TP* is the number of real OCS points in OCS extraction; *TN* is the number of real non-OCS points in non-OCS extraction; *FP* is the number of non-OCS points wrongly classified as OCS points in OCS extraction; *FN* is the number of OCS points wrongly classified as non-OCS in OCS extraction.

#### 4.2.1. Classification Accuracy Evaluation

Through manual classification, the correct number of points of the contact cable, return current cable, and catenary cable is counted and compared with the number of points obtained by the experimental classification algorithm in this paper. The values of *TP*, *TN*, *FP*, and *FN* of the point clouds with a length of 50 m and 100 m are calculated, and then the corresponding precision indexes are calculated through the above four precision evaluation parameter formulas, as shown in Table 2, Table 3 and Table 4.

It can be seen from Table 3 that the average accuracy of classification of the 50 m OCS point cloud with the proposed classification algorithm is 99.83%, the average precision is 100.00%, the average recalls is 99.16%, and the average overall accuracy is 99.58%. 

As shown in Table 4, the average accuracy of classification of the 100 m OCS point cloud with the proposed classification algorithm is 99.89%, the average precision is 99.97%, the average recall is 99.42%, and the average overall accuracy is 99.69%. The classification effect of the 100-m-long OCS point cloud is similar to that of the 50-m-long OCS point cloud data.

This paper used a scale adaptive feature classification algorithm combined with the DBSCAN classification algorithm considering characteristics of the OCS to classify the test point clouds of OCS, and all indicators show that it obtains good experimental results.

#### 4.2.2. TerraSolid Software Processing Results

**Figure 18:**
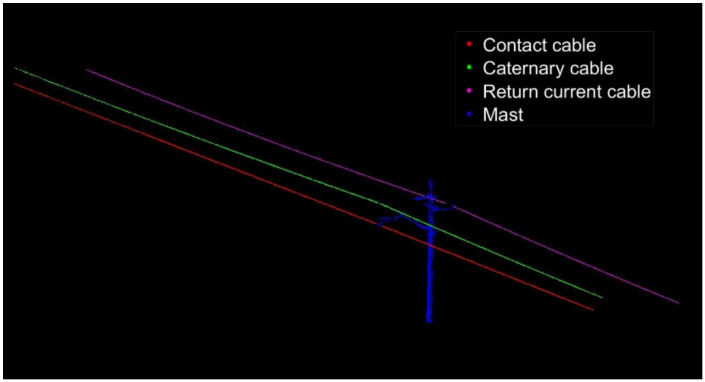
The result of 50 m OCS point cloud classification by TerraSolid.

**Figure 19:**
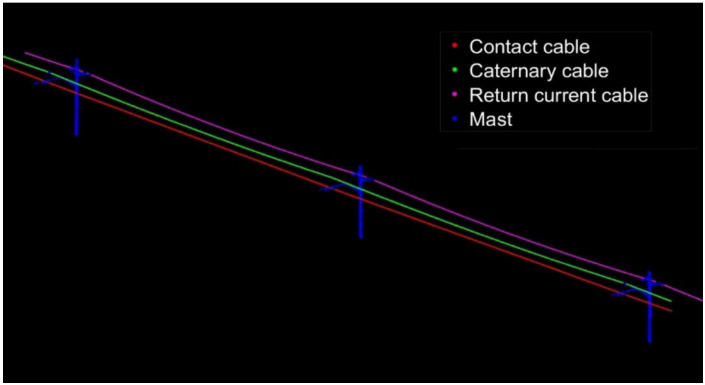
The result of 100 m OCS point cloud classification by TerraSolid.

In order to verify the accuracy and reliability of the algorithm proposed in this article, we use TerraSolid point cloud processing software to classify the two sets of OCS point clouds in this paper and compare the results of the two methods. The result of TerraSolid processing is shown in Figure 18 and Figure 19, respectively.

After statistics, the values of *TP*, *TN*, *FP*, and *FN* are calculated, and then the corresponding accuracy indicators are calculated by the above four accuracy evaluation parameter formulas. The indicators are shown in Table 5 and Table 6.

Compare the accuracy indexes of OCS cables extracted by the methods in this paper with the accuracy indexes of OCS cables extracted by Terrasolid. The results are shown in Table 7.

For the 50-m-long OCS point cloud, the average accuracy of Terrasolid and the algorithm proposed in this paper to extract OCS is 99.80% and 99.83%, respectively; the average precision is 99.99% and 100.00%, respectively; the average recalls are 99.03% and 99.16% respectively; and the average overall accuracy is 99.51% and 99.58% respectively. Obviously, for this set of data, the accuracy of these two methods is very good, and the difference is low.

For the 100-m-long OCS point cloud, the average accuracy of Terrasolid and the algorithm proposed in this paper to extract OCS is 99.81% and 99.89%, respectively; the average precision is 99.99% and 99.97%, respectively; the average recalls are 98.92% and 99.42% respectively; the average overall accuracy is 99.45% and 99.69% respectively. The results show that the algorithm proposed in this paper and Terrasolid used to extract OCS have achieved good classification results and high accuracy. In the average of the four accuracy indicators, except for the precision, the algorithm proposed in this paper is better than the Terrasolid. The extraction accuracy of the catenary cable and the return line is equivalent, but as shown in Table 7, the overall accuracy of the Terrasolid for extracting the OCS is 98.98%, which is significantly lower than that of the contact cable and the return current cable. In conclusion, the method proposed in this paper is more effective in extracting the OCS.

### 4.3. Discussion

In the process of rough classification, the result of OCS by the scale adaptive classification algorithm is not very satisfactory because a few of the points of the mast and cantilever are also considered to be in a linear distribution. Therefore, in order to achieve better classification effects and remove most non-target points, the point cloud is processed multiple times. When the difference between the number of points of the last two iterations is less than 10, we stop the iteration.

Due to the point density or the data quality, the cluster of the same linear object will be discontinuous. The clustering method based on normal is used to merge the discontinuous sub-clusters. The specific process is to traverse all clusters, find the position of two endpoints of each cluster and obtain its direction vector, then classify the two sub-clusters with a similar distance between endpoints and smaller angle of direction vector into one cluster. 

The algorithm proposed in this article needs to use the geometric structure characteristics of the OCS, and the automatic classification of OCS can be realized after inputting the prior structure information of OCS. Terrasolid is a semi-automatic extraction software. As far as the experimental data in this article is concerned, each OCS cable extracted by Terrasolid software requires a manual drawing of the centerline of masts, which may be because the contact mast is not the symmetrical center of the cables, and this process will significantly increase the time.

This paper focuses on the point cloud of the overhead contact system, which has a single structure and no complex background and is only affected by data noise. Therefore, the effectiveness of the algorithm proposed in this article can be guaranteed. This article does not consider OCS extraction in the tunnel and multi-track railways. The situation in the tunnel and multi-track railways is relatively more complicated. 

## 5. Conclusions

The vehicle-mounted LiDAR technology has the advantages of high efficiency and high precision, which can solve the problems of difficulty, low efficiency, and high risk of railway OCS detection. In this paper, experiments were carried out for the classification of OCS from vehicle-mounted LiDAR data. The algorithm proposed in this paper extracted contact cables, catenary cables and return current cables from the OCS point cloud accurately. The average overall accuracy of the method in this paper to extract OCS point clouds of 50 m and 100 m is 99.58% and 99.69%, respectively, which is better than Terrasolid (99.51% and 99.45%, respectively).

The comparison experiment verifies the accuracy and effectiveness of the method. This article focuses on in-depth research on the segmentation and classification algorithm of the OCS point cloud. The main research results and conclusions obtained were as follows:

1. Under the current research background, the research on overhead contact system detection was analyzed. At present, the extraction of OCS from point clouds mainly relies on the structural characteristics of the power line and its positional relationship with the track, and the clustering method is mainly used. This process will bring more errors, and it is necessary to set thresholds when extracting the trackbed and the OCS, which is more subjective. For catenary cables with large curvatures, the effect is often poor. This article analyzed the characteristics of the OCS, and on this basis, put forward the main research content of this article to make full use of and extract the spatial distribution characteristics of target points and improve the accuracy of classification extraction.
2. To classify most of the OCS point cloud in the non-ground point cloud, this paper used the geometric structure feature (dimensional feature) of the point cloud with different scales as the basis for classification. We adopted a scale adaptive feature classification algorithm and introduced the concept of entropy feature values to better select the best scale. We then completed the rough classification of the point cloud; this laid a good foundation for the subsequent classification.
3. Based on the rough classification, this paper adopted a DBSCAN algorithm that takes into account the characteristics of the OCS to realize the fine classification of the OCS point cloud. This algorithm combined the traditional DBSCAN algorithm with the spatial characteristic information of the OCS to achieve the purpose of accurate classification. This method has strong pertinence to the classification of the overhead contact system and provided a new method for the classification of railway OCS from the railway point cloud.

However, the proposed method still has defects and can be improved. The accuracy of the method is affected by the data quality, and the poor data quality may cause more clusters of the same target object. When the environment is more complex, the algorithm may not be able to detect the target object like crossed lines effectively.

In general, this provides a new method for processing railway overhead contact system point clouds, and it has done the basic work for the detection of the railway overhead contact system and also provided the basis for the modeling of the OCS. The method in this paper has high engineering application value in railway component detection. With our further research, we will study the extraction and classification of railway OCS in the relatively complex environment of the tunnel and multi-track railway.

## Figures and Tables

**Table 1:** Technical parameters of German Z + F 3D laser scanner PROFILER 9012.

Parameter Category	Parameter
Scanning system	Z + F PROFILER 9012
Scanning mode	Phase-scanning
Range measure	119 m
Laser grade	1 level
Rotation speed	200 R/s
Angular resolution	0.0088°(40,960 pixel/360°)
Point scan rate	1,016,000 points/s

**Table 2:** TP, TN, FP, and FN values in classification results with the algorithm proposed in this article.

Category		Point Cloud with a Length of 50 m			
		TP	TN	FP	FN
50 m OCS Point Cloud	Contact cable	21,326	98,116	9	234
	Catenary cable	19,027	100,601	8	48
	Return current cable	21,520	98,066	0	90
100 m OCS Point Cloud	Contact cable	7254	25,082	0	75
	Catenary cable	5988	26,347	0	76
	Return current cable	7170	25,224	0	17

**Table 3:** Accuracy evaluation index of classification results of the 50 m OCS point cloud.

Category	Accuracy (%)	Precision (%)	Recall (%)	Overall Accuracy (%)
Contact cable	99.77%	100.00%	98.98%	99.49%
Catenary cable	99.77%	100.00%	98.75%	99.37%
Return current cable	99.95%	100.00%	99.76%	99.88%
Average	99.83%	100.00%	99.16%	99.58%

**Table 4:** Accuracy evaluation index of classification results of the 100 m OCS point cloud.

Category	Accuracy (%)	Precision (%)	Recall (%)	Overall Accuracy (%)
Contact cable	99.80%	99.96%	98.91%	99.43%
Catenary cable	99.95%	99.96%	99.75%	99.85%
Return current cable	99.92%	100.00%	99.58%	99.79%
Average	99.89%	99.97%	99.42%	99.69%

**Table 5:** Accuracy evaluation index for the 50 m OCS point cloud classification results by Terrasolid.

Category	Accuracy (%)	Precision (%)	Recall (%)	Overall Accuracy (%)
Contact cable	99.78%	100.00%	99.04%	99.52%
Catenary cable	99.74%	100.00%	98.61%	99.30%
Return current cable	99.87%	99.97%	99.44%	99.71%
Average	99.80%	99.99%	99.03%	99.51%

**Table 6:** Accuracy evaluation index for the 100 m OCS point cloud classification results by Terrasolid.

Category	Accuracy (%)	Precision (%)	Recall (%)	Overall Accuracy (%)
Contact cable	99.87%	100.00%	99.26%	99.63%
Catenary cable	99.92%	99.99%	99.51%	99.75%
Return current cable	99.63%	99.97%	98.01%	98.98%
Average	99.81%	99.99%	98.92%	99.45%

**Table 7:** Accuracy comparison of the two methods.

Category		The Algorithm in This Paper		Terrasolid	
		50 m Data	100 m Data	50 m Data	100 m Data
Average	Accuracy (%)	99.83%	99.89%	99.80%	99.81%
	Precision (%)	100.00%	99.97%	99.99%	99.99%
	Recall (%)	99.16%	99.42%	99.03%	98.92%
	Overall accuracy (%)	99.58%	99.69%	99.51%	99.45%
Overall accuracy	Contact cable	99.49%	99.43%	99.52%	99.63%
	Catenary cable	99.37%	99.85%	99.30%	99.75%
	Return current cable	99.88%	99.79%	99.71%	98.98%

## Data Availability

Not applicable.
